# A Novel Micro-Displacement Sensor Based on Double Optical Fiber Probes Made through Photopolymer Materials

**DOI:** 10.3390/ma13235475

**Published:** 2020-12-01

**Authors:** Fuzheng Zhang, Qijing Lin, Liangquan Zhu, Na Zhao, Feng Han, Libo Zhao, Zhuangde Jiang

**Affiliations:** 1 State Key Laboratory for Manufacturing Systems Engineering, Xi’an Jiaotong University, Xi’an 710049, China; xjzfz123@stu.xjtu.edu.cn (F.Z.); zhuliangquan@stu.xjtu.edu.cn (L.Z.); zn2015@stu.xjtu.edu.cn (N.Z.); hanfeng_20625@xjtu.edu.cn (F.H.); libozhao@xjtu.edu.cn (L.Z.); zdjiang@mail.xjtu.edu.cn (Z.J.); 2 Collaborative Innovation Center of High-End Manufacturing Equipment, Xi’an Jiaotong University, Xi’an 710054, China; 3 School of Mechanical and Manufacturing Engineering, Xiamen Institute of Technology, Xiamen 361021, China; 4 Xi’an Jiaotong University Suzhou Institute, Suzhou 215123, China

**Keywords:** micro-displacement sensor, optical fiber probe, photopolymerization

## Abstract

In this paper, a novel micro-displacement sensor with double optical fiber probes is proposed and designed, which can realize the highly sensitive sensing of longitudinal or lateral micro-displacements. The optical fiber probes are made through photopolymer formulation, and the effects of reaction time and optical power on the growth length of the probe are illustrated. The relationship between light intensity and longitudinal micro-displacement is a power function in the range of 0–100 μm at room temperature with a correlation coefficient of 98.92%. For lateral micro-displacement, the sensitivity is −2.9697 dBm/μm in the range of 0–6 μm with a linear fit of 99.61%. In addition, the linear correlation coefficient decreases as the initial longitudinal distance increases, and the function of these correlation coefficients is also linear with a linearity of 96.14%. This sensor has a simple manufacturing process, low cost, high sensitivity, and fast response speed. It is suitable for harsh environments such as strong electromagnetic interference and corrosivity, and has a broad application prospect in the field of micro-displacement sensing.

## 1. Introduction

Displacement is an important characteristic parameter in industrial products. At present, with the continuous development of manufacturing technology, the requirements for processing accuracy and technical indicators are getting increasingly higher [1], so a faster and more flexible measurement method that can directly achieve the nondestructive product quality inspection during the production process is needed. The optical fiber sensor has the characteristics of small size, strong adaptability, anti-electromagnetic interference, and high sensitivity [2,3], which play significant roles in solving the problem of micro-displacement measurement in micro-processing and applied physics. For the application of optical fiber in the field of micro-displacement sensing, Fan et al. [4] proposed a method for detecting micro-displacement by using fiber end-face microsphere coupling; however, this sensor can only measure the longitudinal micro-displacement, and the sensitivity is low—only 0.00401 dBm/μm. On the basis of the embedded photonic-crystal-fiber (PCF) modal-interferometer, Dong et al. [5] designed a micro-displacement sensor, but the structure is complicated and the -sensitivity is also low—only 0.0024 dBm/μm. Bao et al. [6] demonstrated a compact fiber-optic quasi-Michelson interferometer (QMI) for micro-displacement measurement. Its sensitivity is high, but its structure is complex, and its displacement sensing range is small. Zhu et al. [7] proposed and demonstrated a novel optical-fiber micro-displacement sensor based on surface plasmon resonance (SPR) by fabricating a Kretschmann configuration on graded-index multimode fiber (GIMMF). This micro-displacement fiber sensor has a high sensitivity with a maximum of up to 10.32 nm/μm, but its detection range is only from 0 to 25 μm, and it can only realize longitudinal micro-displacement detection. In the field of micro-displacement sensing, researchers have also proposed many other structures based on optical fibers [8,9,10,11]. However, almost all research only focuses on single-direction micro-displacement sensing and rarely involves multidirection sensing, which severely limits the application range of optical fiber displacement sensors.

In this paper, a new micro-displacement sensor using the structure of double probes is proposed and designed. On the premise of having the advantages of simple structure and high sensitivity, this sensor can realize micro-displacement sensing in the longitudinal or lateral directions respectively, which provides a good reference for the research of multidirectional micro-displacement sensing. For the probe, a free-radical photopolymerizable formulation is used to generate a cone structure at the end of the ordinary optical fiber, which is also a novel application of this structure in the field of micro-displacement sensors.

## 2. Materials and Methods

### 2.1. Preparation of Optical Fiber Probe

Optical fiber probes are made based on the principle of photopolymerization. Under the action of appropriate light, some photopolymer materials will change from liquid to solid, which is of great significance for the development of optics and photonics [12,13]. There are three components to make up the photopolymerizable formulation: eosin Y (0.5 wt.%), methyldiethanolamine (8 wt.%), and pentaerythritol triacrylate (91.5 wt.%) [14]. Such a system is sensitive between 450 and 550 nm and gets converted through a free-radical cross-linking polymerization under green light irradiation [15]. Specifically, although this formulation has been proposed, further experimental research is carried out, especially the effect of photopolymerization time and optical power on probe generation.

**Figure 1:**
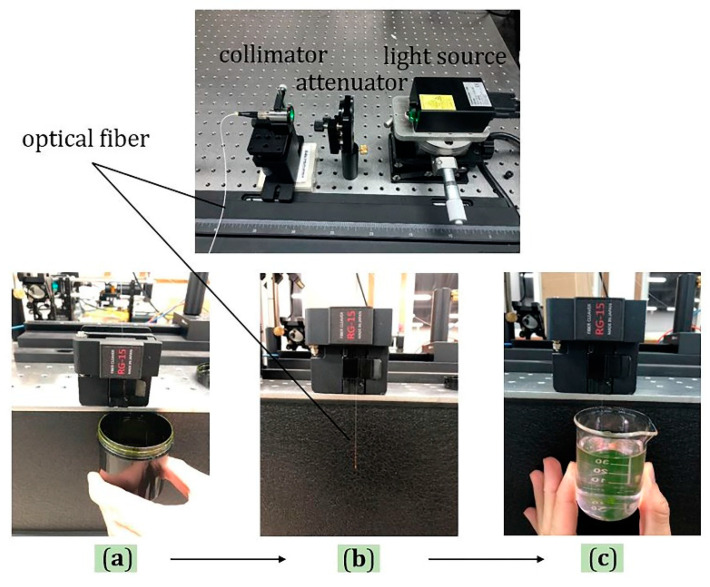
The experimental platform and process for fabrication of optical fiber probes. (**a**) Immerse the end of fiber in the photopolymerizable formulation; (**b**) photopolymerization time; (**c**) use ethanol to remove the surplus photopolymerizable formulation at the end of the fiber.

The experimental platform for fabrication of optical fiber probes was designed and constructed with the advantages of simple structure and convenient disassembly and assembly, as shown in Figure 1, including three parts: the light source, optical attenuator, and collimator. Among them, a laser emitting at 532 nm was used as a light source. The time of the photopolymerization reaction was controlled by controlling the time interval of the light source on and off (that is, the growth time of the fiber probe). In addition, the optical power was controlled by adjusting the attenuator, and finally, the light was coupled into the fiber through the collimator.

**Figure 2:**
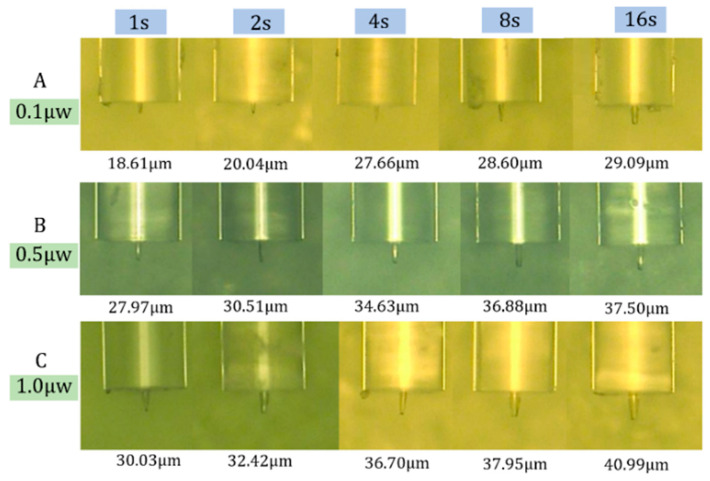
The manufactured optical fiber probes: (**A**): *p* = 0.1 μW, (**B**): *p* = 0.5 μW, (**C**): *p* = 1.0 μW.

**Figure 3:**
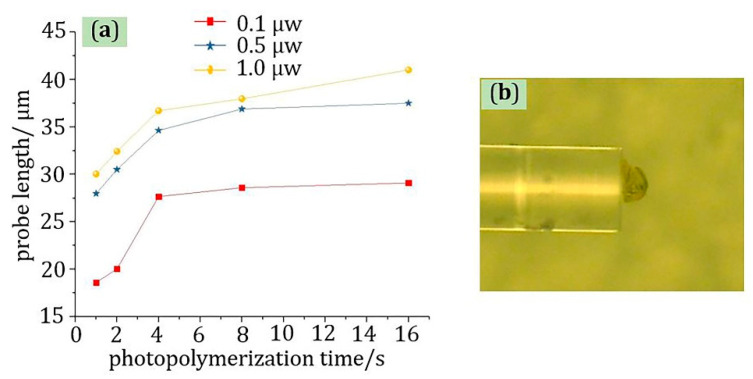
The experimental analysis results: (**a**) the curve of the relationship between probe length and time; (**b**) physical picture of cluster phenomenon caused by high power.

The manufactured optical fiber probes with different optical power and photopolymerization reaction time are shown in Figure 2; there were three sets of experiments in total; the optical power was 0.1, 0.5, and 1.0 μW in order; and the time was 1, 2, 4, 8, and 16 s, respectively. It can be seen from the experimental results that the length of the probe increased with the increase in the optical power and exposure time, respectively. Furthermore, as shown in Figure 3a, the growth rate of the probe decreased with increasing exposure time, and the inflection point of time was about 4 s. In addition, when the power of the light source is too large, clusters at the end of the optical fiber are easily caused. The shape of the probe produced with a light source power of 10 μW is shown in Figure 3b. The cause of this phenomenon may be that the instantaneous energy at the end of the fiber was too large due to the large power, which caused a large area of photopolymerization to occur in a short period of time.

### 2.2. Principle of Micro-Displacement Sensing

**Figure 4:**
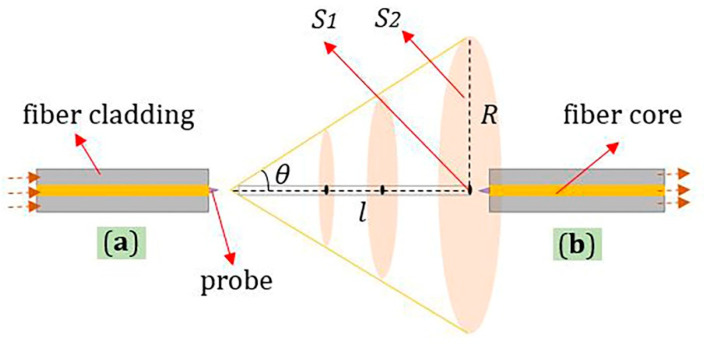
The principle of longitudinal micro-displacement (Left-Right): (**a**) optical input fiber probe; (**b**) optical receiving fiber probe: *S_1_* is the area of received light spot, *S_2_* is the area of incident light spot, *θ* is half of the incident angle, *R* is the radius of the *S_2_*, *l* is the distance between two probes.

The light is divergent at the output end of an optical fiber. The polymer tip can be viewed as an extension of the fiber core [16]. The sensing principle of the sensor’s longitudinal micro-displacement is shown in Figure 4; *S_1_* is the area of received light spot, *S_2_* is the area of incident light spot, and *l* is the distance between two probes. *S_1_* was unchanged, and *S_2_* increased as *l* increased. The ratio of *S_1_* to *S_2_* can approximate the relationship between the optical power received by the probe and the longitudinal displacement.

(1) $$S_{2}=\pi R^{2}=\pi (l\tan\theta {)}^{2}=\pi \tan^{2}\theta l^{2}$$

(2) $$P=P_{0}\frac{S_{1}}{S_{2}}=P_{0}\frac{S_{1}}{\pi \tan^{2}\theta l^{2}}=Cl^{-}{}^{2},C=\frac{P_{0}S_{1}}{\pi \tan^{2}\theta }$$

where *C* is a constant, *P* is the optical power received by fiber optic probe, and *P_0_* is the total optical power of the fiber output. Thus, the magnitude of the received optical power is a power function relation of the distance of longitudinal micro-displacement.

**Figure 5:**
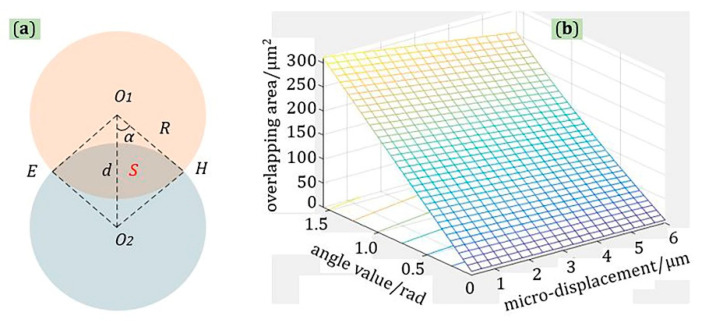
The principle of lateral micro-displacement (Up–Down): (**a**) schematic diagram; (**b**) relationship diagram of angle value-micro-displacement-overlapping area: *S* is the overlapping area of incident light spot and received light spot, *α* is half of the angle, *R* is the radius of the light spot, *d* is the value of lateral micro-displacement.

As shown in Figure 5a, for the lateral micro-displacement, because the longitudinal distance between the two probes was constant, the size of the incident light spot and the received light spot were equal, and the received optical power of the probe could be approximately estimated by calculating their intersection area. Equation (3) is the area of sector *OEO_1_H* and sector *OEO_2_H*, and they are equal. Equation (4) is the area of quadrilongitudinal □*O_1_EO_2_H*, and Equation (5) represents the overlapping area of incident light spot and received light spot.

(3) $${\overset{\wedge }{S}}_{○}{}_{EO_{1}H}={\overset{\wedge }{S}}_{○EO_{2}H}=\frac{2\alpha }{2\pi }\pi R^{2}=2R^{2}\alpha$$

(4) $${\overset{\wedge }{S}}_{□}{}_{O_{1}EO_{2}H}=2{\overset{\wedge }{S}}_{△O_{1}O_{2}H}=Rd\sin\alpha$$

(5) $$S={\overset{\wedge }{S}}_{○}{}_{EO_{1}H}+{\overset{\wedge }{S}}_{○EO_{2}H}-{\overset{\wedge }{S}}_{□}{}_{O_{1}EO_{2}H}=2R^{2}\alpha -Rd\sin\alpha$$

where *R* is a fixed value, and the range of *α* is from 0 to *π/2*. Thus, as shown in Figure 5b, after calculation and analysis using MATLAB software, it was found that no matter what the value of *α* is, the relationship between *S* and *d* is a linear function.

## 3. Results and Discussion

**Figure 6:**
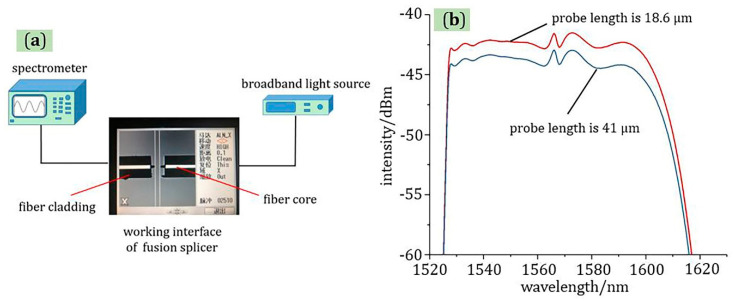
(**a**) The test principle of micro-displacement sensor with double fiber probes; (**b**) transmission spectrum of different probe lengths.

The test principle of micro-displacement sensor is shown in Figure 6; both optical fiber probes are fixed in the optical fiber fusion splicer, and the tips of two probes are on the same longitudinal line by adjusting the parameters of the fusion splicer. The existence of the probe is very important to the micro-displacement sensor, which improves the coupling efficiency of light. Compared with the fiber end-face displacement sensor, the micro-displacement sensor with robe structure had higher sensitivity because of the high coupling efficiency, Furthermore, as shown in Figure 6b, the red line represents the transmission spectrum of a sensor with the probe length of 18.6 μm, and the blue line represents the transmission spectrum of a sensor with the probe length of 41 μm. The same light source was used in the experiment. At the same coupling distance, a high power received at the receiving end corresponded to a high coupling efficiency. It can be concluded from the experimental results that the shape of the affects the performance of the sensor. The shorter the probe, the higher the coupling efficiency of the sensor. Therefore, the length of the optical fiber probe used in the experiment was about 18.6 μm, and the diameter was about 9 μm. In addition, the two probes were connected to a broadband source (C + L band) and a spectrograph, respectively. The change of longitudinal and lateral micro-displacement can be controlled by adjusting the fusion splicer. An extremely small change of displacement can cause a change for the light intensity of the fiber probe connected to the spectrometer, which can achieve the purpose of detecting longitudinal or lateral micro-displacement by analyzing the received spectrum.

**Figure 7:**
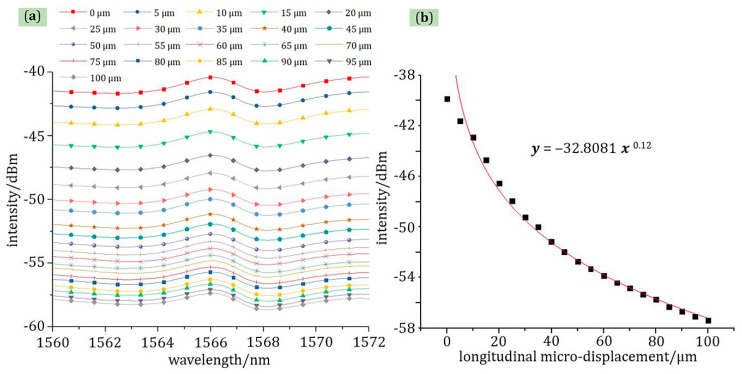
The test curve of longitudinal micro-displacement: (**a**) the relationship of wavelength-light intensity; (**b**) the relationship of micro-displacement-light intensity at 1566 nm wavelength.

As shown in Figure 7, the relationship between light intensity and longitudinal micro-displacement was a power function in the range of 0–100 μm at room temperature with a correlation coefficient of 98.92% at 1566 nm wavelength, which is consistent with theoretical calculations. The displacement interval for each test was 5 μm. After the longitudinal displacement exceeded 100 μm, as the micro-displacement changed, the light intensity change was slight. In addition, as shown in Figure 7a, from 1560 to 1572 nm, the change range of light intensity and micro-displacement was constant—that is, the sensitivity of the longitudinal micro-displacement cannot be affected by the signal light of different wavelengths, which also applies to lateral micro-displacement sensing.

Equation (6) is the relationship between light intensity and longitudinal micro-displacement after converting “dBm” to “W”. Compared with the results of the theoretical analysis, the trend of experimental test results of longitudinal micro-displacement changes is the same, which shows the correctness of the theoretical model. However, the power law of theoretical analysis is not exactly the same as the power law of experimental results—that is, the power of the independent variable in Equation (6) is larger than in Equation (2). The reason for this phenomenon is that the divergent beam of the probe cone and the divergent beam at the junction of the probe structure and the fiber core are ignored in the theoretical model, so the power value of the experimental results is larger than the power value of the theoretical analysis.

(6) $$y=3.5871x^{-0.97}$$

**Figure 8:**
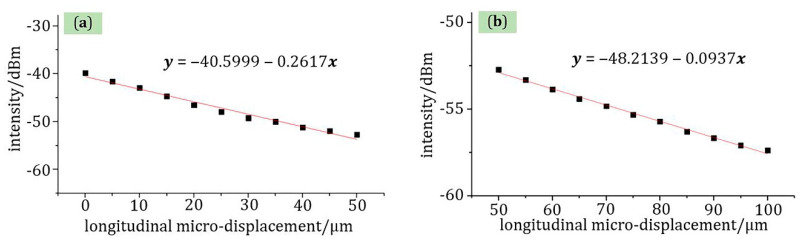
The relationship of micro-displacement-light intensity at 1566 nm wavelength (**a**) the range of 0–50 μm and (**b**) the range of 50–100 μm.

Moreover, the relationship between light intensity and longitudinal micro-displacement was also a piecewise linear function. As shown in Figure 8, the sensitivity of the sensor was −0.2617 dBm/μm in the range of 0–50 μm at room temperature with a linear fit of 98.06%, and from 50 to 100 μm, the sensitivity was −0.0937 dBm/μm with a linear fit of 99.54%. For this sensor structure, the decisive role in micro-displacement sensing is the polymer tip. Although the new fiber probe tip is small in size, it has high optical coupling efficiency. Therefore, the micro-displacement measurement range of the sensor is large, which can reach 100 μm.

**Figure 9:**
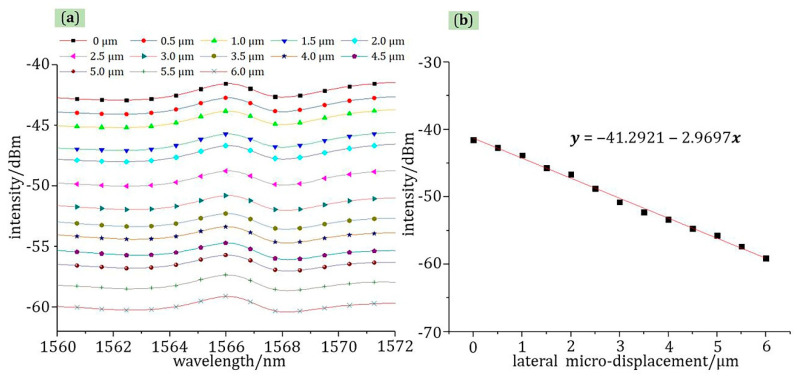
The test curve of lateral micro-displacement: (**a**) the relationship of wavelength-light intensity; (**b**) the relationship of micro-displacement-light intensity at 1566 nm wavelength.

For the measurement of lateral micro-displacement, as shown in Figure 9, the relationship between displacement and light intensity was linear in the range of 0–6 μm at room temperature, which is consistent with theoretical calculations. The displacement interval for each test was 0.5 μm. The correlation coefficient and sensitivity were 99.61% and −2.9697 dBm/μm at 1566 nm wavelength, respectively.

**Figure 10:**
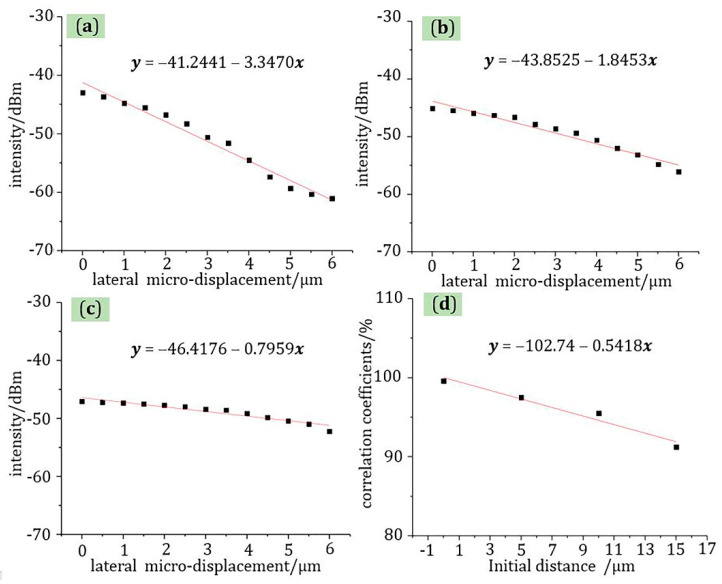
The test curve of lateral micro-displacement at 1566 nm wavelength: (**a**) the initial distance is 5 μm; (**b**) the initial distance is 10 μm; (**c**) the initial distance is 15 μm; (**d**) function curve between initial distance and correlation coefficient.

In addition, for lateral micro-displacement, micro-displacement sensing experiments with initial distances of 5, 10, and 15 μm were also tested. The test environment was also at room temperature, and the range of lateral displacement was from 0 to 6 μm. From the test results in Figure 10, the function relationships of micro-displacement-light intensity in these three cases were all linear, and their correlation coefficients were 97.50%, 95.52%, and 91.24%, respectively. In addition, combined with the test results in Figure 9 introduced above, their respective correlation coefficients had a tendency to decrease as the initial distance increased. The reason for this phenomenon is that the propagation of light in the air can cause energy loss. Therefore, as the initial distance increases, the energy loss increases, and the measurement error of the sensor also increases, which causes increased data dispersion. Therefore, the correlation coefficient of lateral displacement sensing decreases with the increase of initial distance. As shown in Figure 10d, the function of these correlation coefficients was also linear with a linear fit of 96.14%, which shows that the decrease in the fitted correlation coefficient was regular.

## 4. Conclusions

The micro-displacement sensor with double optical fiber probes proposed in this paper not only has the advantages of simple structure, high sensitivity, and easy operation, good linear fit, etc., but more importantly, it can realize longitudinal or lateral micro-displacement sensing, respectively, which is also the biggest advantage of the sensor. In summary, the relationship between the length of the probe and the parameters such as the reaction time and the optical power are found through photopolymerization experiments. This new type of high-sensitivity sensor will inevitably have great application value in the field of micro-displacement detection. Moreover, based on the high coupling efficiency to light, the fiber probe structure may also have important application value in the fields of strain and angle measurement.

In the future, we plan to solve the problem of simultaneous measurement of micro-displacement of the sensor in two directions through factorial design. The use of factorial design is a powerful tool that provides a considerable amount of information with a minimum number of experiments [17], which is very helpful for solving the problem of measuring micro-displacement in two directions at the same time. Furthermore, this sensor can be used with other fiber optic sensors such as twist/torsion sensors [18] to provide complementary physical information, which further improve the application value of the sensor.
